# Cytotoxicity of Self-Adhesive Resin Cements on Human Periodontal Ligament Fibroblasts

**DOI:** 10.1155/2018/7823467

**Published:** 2018-11-29

**Authors:** Fangfang Sun, Ying Liu, Yahui Pan, Meng Chen, Xiangfeng Meng

**Affiliations:** ^1^Department of Prosthodontics, Nanjing Stomatological Hospital, Medical School of Nanjing University, Nanjing, China; ^2^Department of Stomatology, The Affiliated Jiangning Hospital of Nanjing Medical University, Nanjing, China; ^3^Department of Respiratory, Children's Hospital of Nanjing Medical University, Nanjing, China

## Abstract

The aim of this study was to evaluate the potential cytotoxicity of self-adhesive resin cements with or without light irradiation on human periodontal ligament fibroblasts (HPDLFs)* in vitro*. Three self-adhesive resin cements (RelyX U200, Maxcem Elite and Multilink Speed) were cured with light or not. Cured cements were stored at 37°C for 24 h in water or Dulbecco's modified eagle medium (DMEM) medium. Their chromatographic analysis of water-based extract solution was made and then the DMEM-based extract solution was diluted in complete DMEM {1:5, 1:10, 1:20, 1:40, 1:80 (v/v)} for evaluating cell relative growth rate and cell apoptosis/necrosis rate of HPDLFs. The data was analyzed by one-way ANOVA and independent T test. Regardless of light irradiation, cell relative growth rate increased, and the apoptosis/necrosis rate of each resin cement decreased with the increase of gradient dilution. Regardless of gradient dilution, the cell relative growth rate and apoptosis/necrosis rate of RelyX U200 and Maxcem Elite with light irradiation were higher than those without light irradiation. Besides, without light irradiation, Multilink Speed showed higher cell relative growth rate and lower apoptosis/necrosis rate than other cements. Light irradiation and composition difference of self-adhesive resin cements could affect their cytotoxicity on HPDLFs.

## 1. Introduction

Self-adhesive resin cements, defined as cements based on filled polymers designed to adhere to tooth structure without the requirement of a separate adhesive or etchant, were introduced to dentistry within the past decade but have gained rapidly in popularity. The incorporation of acid functional monomer is a critical component in self-adhesive resin cements, which could not demineralize/dissolve the smear layer completely to obtain mechanical retention [1, 2], while could form effective chemical bonding to the tooth by the acid-base neutralization reaction like glass ionomer cement [3]. According to some* in vitro *studies, self-adhesive resin cements had satisfied effects comparing to other multistep resin cements [4]. However, because of the low number of studies available, the clinical evidence of self-adhesive cements cannot be assessed in sufficient manners [4, 5]. In addition to adhesion, other properties of self-adhesive resin cements such as solubility, absorption, and polymerization capability, could be related to their clinical behaviors.

The concentration of the acidic monomers in the self-adhesive resin cements should be sufficiently high to achieve an acceptable bonding to the dentin and enamel; meanwhile, as the acid functionality is consumed through reaction with calcium on the tooth and a variety of metal oxides from the ion-leachable filler, these materials become more hydrophobic [3]. However, studies have indicated that most self-adhesive resin cements exhibited higher sorption and solubility than conventional resin cements [6–10]. The excessive hydrophilic character of self-adhesive resin cement can cause swelling that in turn can compromise surface dimensional stability [9–12].

Compared with conventional resin materials, self-adhesive resin cements containing acidic monomers have a more complex polymerization process. The dominant setting reaction is the radical polymerization which can be achieved by light exposure or through the self-curing mechanism resulting in cross-linking polymerization of monomers [3]. Besides, ions released from the acid-soluble fillers can neutralize the acidic groups to create a chelate reinforced three-dimensional methacrylate network [3]. However, in redox polymerization, benzoyl peroxide is susceptible to radical formation under the low pH conditions present in the acidic component of the self-adhesive resin cements, and amines form salts that greatly decrease their activity with peroxides even more so than in the case of amines used as photoreductants with a photosensitizer [13]. Moraes et al. [14] suggested that self-adhesive resin cements may present slower rate of polymerization and lower final polymerization degree than conventional resin cements, in either the dual- or self-cured mode, and the final polymerization degree of self-adhesive resin cements under dual-cure mode was generally higher than that under self-cure mode. And Yoshida et al. [15] also found the hardness of self-adhesive resin cements depended on the depth of the cavity, namely, the amount of light irradiation. And the barrier of composite and ceramic shielding the light irradiation could significantly decrease the polymerization degree of dual-cured self-adhesive resin cement [16–18].

The self-adhesive resin cements exhibit the hydrophilic character and the polymerization ability depended on the light irradiation, which might cause more free acid, polymeric intermediate product, and other components to dissolve and release in saliva. To date, only a limited number of studies have reported the cytotoxicity of self-adhesive resin cements. Nocca et al. [16] showed that the amount of monomers released from the cured self-adhesive resin cements in the absence of barriers was significantly lower than that released in the presence of either the ceramic or the composite barrier, which induced slight cytotoxicity on human pulp cells. Currently the biocompatible studies of self-adhesive resin cements mainly focused on the pulpal response and suggested that self-adhesive resin cements could induce a slight to moderate inflammatory response, which was inversely related to the remaining dentine thickness [19–22].

While the biocompatibility of self-adhesive resin cements on periodontium also appears as the important evaluation factor in their clinical applications, therefore, the aim of this study was to evaluate the potential cytotoxicity* in vitro *of self-adhesive resin cements with or without light irradiation on human periodontal ligament fibroblasts (HPDLFs).

## 2. Materials and Methods

Three self-adhesive resin cements (RelyX U200-RU, Maxcem Elite-ME and Multilink Speed-MS) were used in this study. Their composite specifications are listed in Table 1.

### 2.1. Specimen Preparation

According to the manufacturer's instructions, disc specimens (5 mm in diameter, 1 mm in thickness) of each resin cement were prepared by using a cylindrical transparent acrylic resin tube. Both sides of some specimens were irradiated by a LED light unit with the intensity of 800 mW/cm^2^ (Bluephase C8, Ivoclar Vivadent, Schaan, Principality of Liechtenstein) for 20 s. And some specimens without light irradiation were self-cured. Cured specimens were stored in light-proof box at 37°C for 24 hours.

### 2.2. Liquid Chromatographic Analysis of Extract Solution

The extraction solutions of cements were prepared according to the ISO10993-12 international standard. Ten specimens were obtained from each group and scattered into the centrifuge tubes. 2.5 mL deionized water was added to sample, in which the value of surface area/liquid medium volume was 3 cm^2^/mL according to the standard. The specimens were soaked at 37°C for 24 h, and the extract solutions were collected. After bacteriological filtration, the water-based extraction was used for the liquid chromatographic analysis by liquid chromatography analyzer (Agilent 1200, Agilent Technologies, Santa Clara, California).

The analysis was carried out by a Kromasil C18 chromatographic column (4.6mm ×250 mm, 5 m) at column temperature of 30°C. The mobile phase was methanol: water (70: 30 in volume ratio) with a flow rate of 1.0 mL/min. The ultraviolet detection wavelength was 254 nm. Sample size was 5 *μ*L and eluting time was 12 min.

### 2.3. Primary Culture and Identification of HPDLFs

Normal noncarious premolars from orthodontic patients (age ranging from 10 to 20 years old) were obtained, and proper informed consent was obtained from these donors. Periodontal membrane was extracted on a super-clean bench. After washing with phosphate buffered saline (PBS) and the periodontal tissue was cut into fragments (size of 1 × 1 × 1 mm^3^) and was placed evenly in a culture dish and then incubated in Dulbecco's modified eagle medium (DMEM ) complete medium under 37°C and 5%CO_2_. The cellular immunochemical fluorescence assay was used to observe the expression of vimentin and keratin on the cells to identify the characteristics of HPDLFs. HPDLFs with logarithmic growth in 3-5 generations were used for cytotoxicity test.

### 2.4. Cell Morphological Observation of HPDLFs

The extraction solutions of cements were prepared according to the ISO10993-12 international standard. Ten specimens were obtained from each group and scattered into the centrifuge tubes. 2.5 mL DMEM complete medium (containing 10% fetal calf serum and 1×DMEM medium of penicillin and streptomycin) was added according to the standard value of 3 cm^2^/mL for sample surface area/liquid medium volume. The specimens were soaked at 37°C for 24 h, and the extraction solutions were collected. The DMEM-based extraction solution was diluted using DMEM complete medium with the volume fraction of 1:5, 1:10, 1:20, 1:40, and 1:80.

1×10^5^/mL HPDLFs were plated on 24 well plates (500 *μ*L/well). After cell adherence, the experimental groups were treated with differently concentrated extraction solutions, while the control groups were treated with normal DMEM medium without extraction solutions. Three compound holes were set for each group. Cells were cultured under the temperature of 37°C and 5%CO_2_ for 24 h and were then examined using a phase contrast microscope.

### 2.5. Cell Relative Growth Rate of HPDLFs Evaluation

1×10^5^/mL HPDLFs were plated on 96 well plates (100 *μ*L/hole). Six compound holes were set for each group. After cell adherence, the experimental groups were treated with DMEM mixed with differently concentrated extraction solutions, while the control groups were treated with normal DMEM medium without extraction solutions. Cells were cultured at 37°C and 5%CO_2_ for 24h. Consequently, cells were washed 2 times with PBS and then incubated with CCK-8/DMEM medium solution (volume ratio of 1:10 (serum-free)) for additional 4 hours. The absorbance value at 450 nm was determined by enzyme labeling instrument. CCK-8/DMEM medium solution without cells was served as Blank groups. The cell relative growth rate (%) was calculated based on the following formula: [A_Experimental  groups_-A_Control  groups_]÷[A_Control  groups_-A_Blank  groups_]×100%.

### 2.6. Cell Apoptosis/Necrosis Rate of HPDLFs Evaluation

1×10^5^/mL HPDLFs were plated on 6 well plates (2 mL/hole). Three compound holes were set for each group. After cell adherence, the DMEM medium without extraction solutions was added to the control groups, and the extraction solutions with different concentrations were mixed into the experimental groups. All groups were cultured for 24h under the temperature of 37°C and 5%CO_2_. After washing with PBS, cells were digested using 0.25% trypsin and collected after centrifugation. The binding buffer was used for the resuspension preparation to achieve the cell suspensions with cell density of 1× 10^7^/mL. 100 *μ*L suspension solution was then retrieved and mixed with Annexin V-FITC/PI dying for 15 min in dark. Then 400*μ*L binding buffer was added to each sample. The flow cytometry was employed to detect cell apoptosis/necrosis rate.

### 2.7. Statistical Analysis

The mean values and standard deviations were calculated for each test group. The data was analyzed by SPSS 20.0 (SPSS Inc., Chicago, Illinois, USA). Independent T test was used to test the difference in cell relative growth rate and apoptosis/necrosis rate of same materials between with and without light irradiation. The one-way ANOVA and SNK test were used to test the cell relative growth rate and apoptosis/necrosis rate among three cements under same gradient dilution or among different gradient dilution for the same cement. The significance was set at the level of 0.05.

## 3. Results

**Figure 1:**
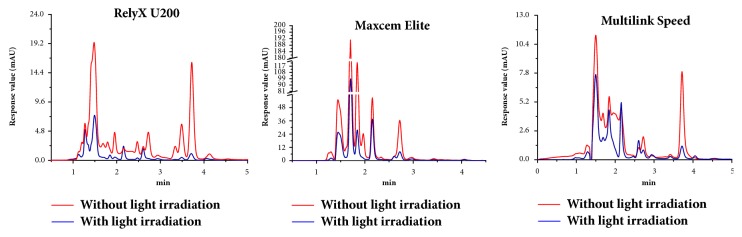
Liquid chromatographic separation analysis of water-based extract solution of three resin cements with or without light irradiation. Ordinate mAU indicated the response values of precipitated materials and abscissa min indicated the precipitation peak time point.

Liquid chromatographic separation analysis of extract solution of three resin cements with or without light irradiation was shown in Figure 1. RU, ME, and MS without light irradiation showed 18, 17, and 14 distinct peaks irrespectively, while RU, ME, and MS with light irradiation showed 13, 14, and 13 distinct peaks irrespectively. And three resin cements without light irradiation showed higher absorption peak value (mAU) than them with light irradiation.

**Figure 2:**
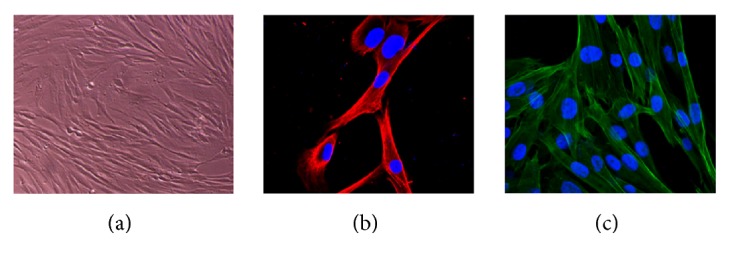
Morphology of human periodontal ligament fibroblast (HPDLFs). (a) Immunofluorescence assay test results (b) Red: Vimentin, Blue: DAPI (c) Green: F-actin, Blue: DAPI, Red: CK18.

The primary cultured HPDLFs were typical fibroblasts with fusiform shape, plump cytoplasm, and clear nucleoli as shown in Figure 2(a). Furthermore, the immunofluorescence test showed positive expression of vimentin as shown in Figure 2(b) and negative expression of keratin as shown in Figure 2(c), which further validated the presence of HPDLFs.

**Figure 3:**
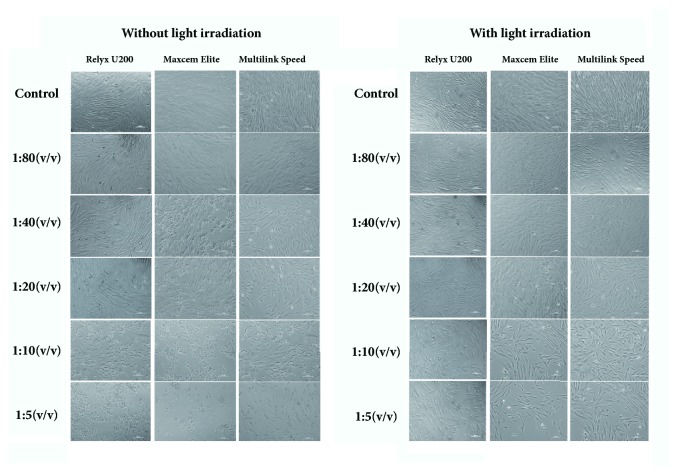
Morphological changes of HPDLFs treated with extract solution of three resin cements with or without light irradiation.

Morphological changes of cells treated with extract solution of resin cements were shown in Figure 3. Without light irradiation, three resin cements showed cell number decrease, cell shrinkage, and filamentous pseudopodia shortening, even though the gradient dilution was 1:80. With light irradiation, ME showed cell shrinkage, particles deposition in the cytoplasm, and cell number decrease when the gradient dilution was less than 1:40, and RU and MS showed obvious cell extension and decrease in the number of cells when the gradient dilution was less than 1:20.

**Figure 4:**
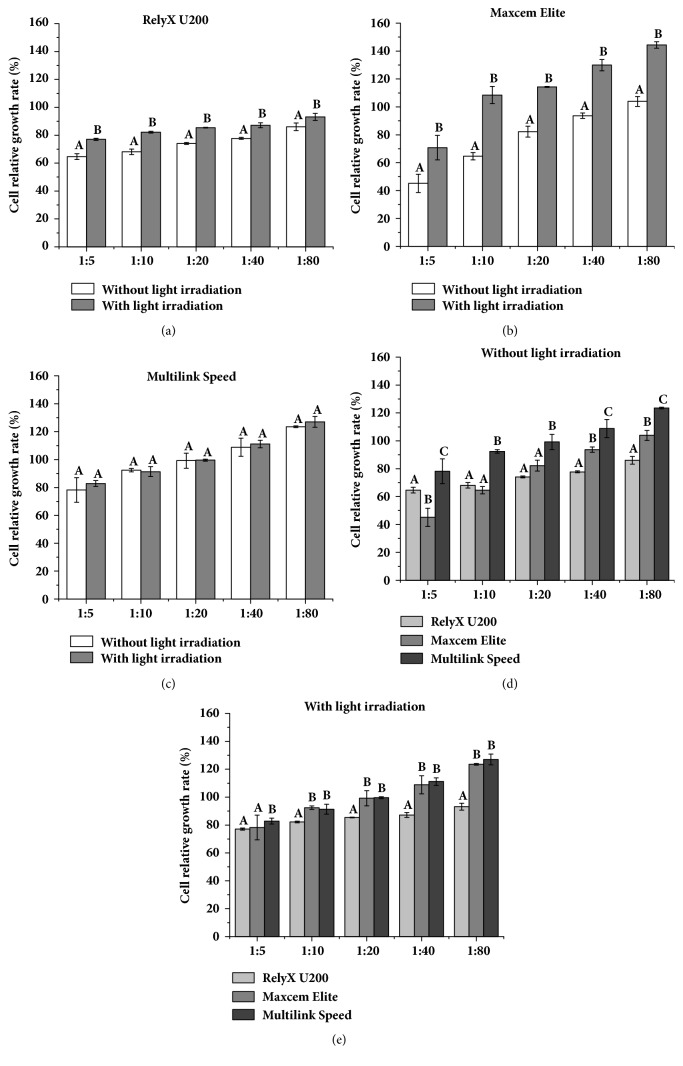
The relative growth rates of HPDLFs treated with extract solution of three resin cements with or without light irradiation. Note: identical uppercase letter indicated that there were no statistical differences between two groups with the same dilution concentration (P>0.05).

HPDLFs' relative growth rates treated with extract solution of three resin cements with or without light irradiation were shown in Figure 4. With the increase of gradient dilution, the cell relative growth rate of each material showed an increasing trend. Under the same gradient dilution, the cell relative growth rates of RU and ME without light irradiation were significantly lower than them with light irradiation, and MS have no difference. Without light irradiation, MS had higher cell relative growth rate than RU and ME.

**Figure 5:**
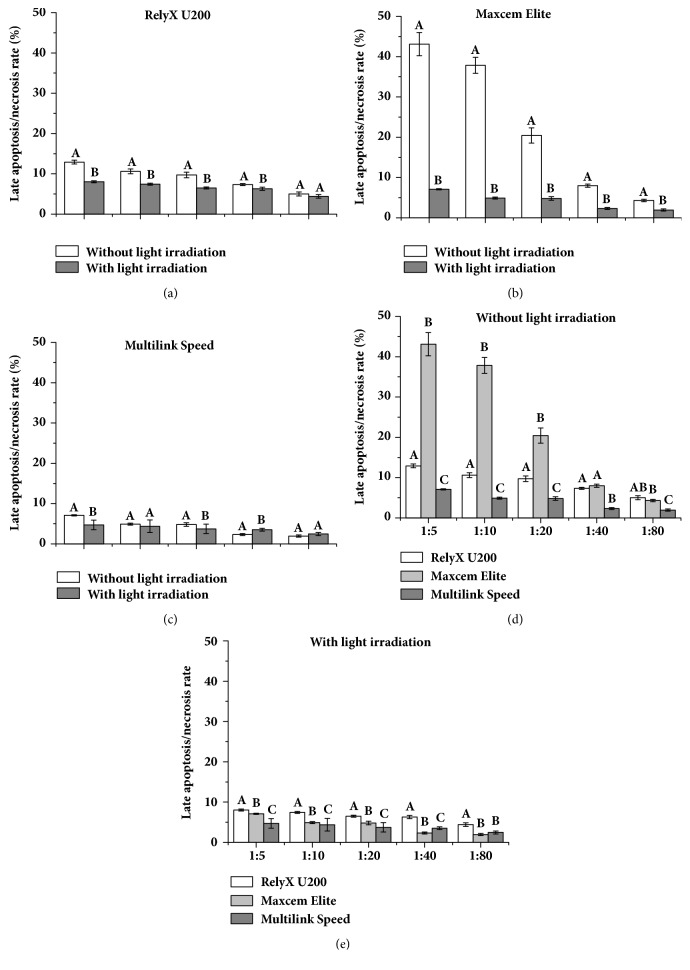
The apoptosis/necrosis rates of HPDLFs treated with extract solution of three resin cements with or without light irradiation. Note: identical uppercase letter indicated that there were no statistical differences between two groups with the same dilution concentration (P>0.05).

HPDLFs' apoptosis/necrosis rates treated with extract solution of three resin cements with or without light irradiation were shown in Figure 5. With the increase in gradient dilution, the cell apoptosis/necrosis rate of each material showed a decreasing trend. Under the same gradient dilution, the cell apoptosis/necrosis rates of RU and ME without light irradiation were significantly lower than them with light irradiation, and MS have no significant difference. Without light irradiation, MS had lower cell apoptosis/necrosis rate than RU and ME.

## 4. Discussion

Many studies shown RU, ME, and MS without light irradiation have lower polymerization degree or hardness than them with light irradiation [14–18], plus their high values of water absorption, solubility, and water expansion stress [6–10], which might lead to a series of changes in extract solution of self-adhesive resin cements. In this study, more distinct absorption peaks and higher absorption peak value occurred in the extract solution of each self-adhesive resin cement without light irradiation. According to some product information provided by the manufacturers, a variety of methacrylate monomer, acid monomer, initiators, and many others could be dissolved out in the water medium [23]. However, it is regrettable that we still could not obtain an accurate reference material to judge the exact substances represented by absorption peaks of the liquid chromatographic analysis. Even so, the result of liquid chromatographic analysis suggested that the insufficient polymerization degree of self-adhesive resin cements without light irradiation produced more dissolving products and species, which might bring higher risk of cytotoxicity.

Previous studies have shown that resin cements significantly reduce the cell viability [24], increase reactive oxygen species (ROS) production [25], and cause cell cycle arrest [26], irrespective of the activation protocol but especially when chemically activated [24]. The study of Ulker et al. [20] showed that RU and ME may modify pulp cell metabolism when the materials are used in deep cavities or directly contact pulp tissue. However, da Fonseca Roberti Garcia et al. [21] indicated that RU can be considered as nontoxic to pulp cells, in accordance with the safe limits of ISO 10993-5:1999 (E) recommendations. And most recent research showed that RU, ME, and MS could significantly reduce the cell viability of immortalized rat odontoblast cells, irrespective of light irradiation, and total death of cells significantly increased when exposed to ME without light irradiation [22], while, in this study, the extract solution of RU, ME, and MS was demonstrated to be cytotoxic to HPDLFs, which could inhibit cell growth and induce cell apoptosis/necrosis. And the cytotoxicity levels on HPDLFs of RU and ME without light irradiation were higher than that with light irradiation, which might be related to the fact that the extract volume without light irradiation was more than twice as high as that with light irradiation as shown in Figure 1. Similar with a previous study [22], ME without light irradiation showed a significantly higher cytotoxicity on HPDLFs at less than 1:40 gradient dilution. In clinical cases, the presence of the ceramic, the composite barrier, and tooth cavity is inevitable, and the resulting polymerization of self-adhesive resin cement is insufficient, which induces that higher cytotoxic damage should be attended.

Although MS without light irradiation also suffered insufficient polymerization and more extract, its cytotoxicity level was not significantly different from that with light irradiation. And without light irradiation, MS had lower cytotoxicity than RU and ME. It indicated that, besides light irradiation, the composition of self-adhesive resin cement might be another important effect factor on cytotoxicity. Researches showed that traditional monomers such as Bis-GMA (Bisphenol A diglycidyl methacrylate), TEGDMA (triethylene-glycoldimethacrylate), acid monomers such as 4-META, MDP, and initiator released from dental resin composites and cements have the cytotoxicity [27–36]. The resin matrix of RU and ME includes Bis-GMA, TEGDMA, and acid monomer, while the resin matrix of MS only contains dimethacrylate and acidic monomers. Ratanasathien et al. [27] reported that Bis-GMA shows the highest toxicity against mouse fibroblasts, followed by UDMA (urethane dimethacrylate), TEGDMA, and HEMA (2-hydroxyethyl methacrylate) in order to decrease cytotoxicity. Previous studies reported that, in addition to induction of apoptosis, Bis-GMA causes significant depletion of intracellular GSH content after 18 hours of incubation in human gingival fibroblasts in vitro [28], stimulated ROS production, prostanoid production, cell cycle arrest, apoptosis, and cell death [29–31]. And TEGDMA was reported to have an adverse effect on cell proliferation and exert proapoptotic and toxic effects on THP-1 cells, a human monocytic leukemia cell line. Furthermore, TEGDMA can cause DNA damage, induce a decrease of intracellular glutathione (GSH) [32–34], and increase cyclooxygenase-2 expression and prostanoids production [35]. Sun et al. [36] suggested that acid monomers such as 4-META and MDP could induce the strong cytotoxic effect on HPDLFs. What is more, not only degree of C=C conversion and monomer-release determine the biocompatibility of adhesives, but also cytotoxicity of the (photo-)initiator should be taken into account. Bart Van Meerbeek et al. [37] reported that addition of diphenyl-(2,4,6-trimethylbenzoyl) phosphine oxid (TPO) rendered a universal adhesive more toxic compared to camphor quinone/amine (CQ); however, this effect could be annulled by a thin dentin barrier.

Based on the result of this study, besides light irradiation condition, the composition of material may cause differential cytotoxic effects and should be considered when selecting self-adhesive resin cement. Since the insufficient polymerization degree produced by self-cure mode is unavoidable, the cytotoxicity of self-adhesive resin cement may be minimized by adjusting the composition of the material.

## 5. Conclusions

The composition and light irradiation of self-adhesive resin cements could affect cell proliferation and cell apoptosis induction of HPDLFs.

## Figures and Tables

**Table 1:** Composition of self-adhesive resin cements tested in this study.

Materials	Composition	Lot No.	Manufacturer
RelyX U200	Resin matrix: triethyleneglycol dimethacrylate, 2-propenoic acid, 2-methyl 1,1′-(1-[hydroxymetil]-1,2-ethanodlyl) ester dimethacrylate, 1-benzyl-5-phenyl-barbic-acid, 1,12-dodecane dimethacrylate, tert-butyl peroxy-3,5,5-trimethylhexanoate	A2 623700	3M ESPE AG, St. Paul, Germany
	Filler: 70wt% content, 12.5*μ*m mean particle size, silanated silica, sodium persulfate,titanium dioxide, calcium hydroxide, sodium p-toluene sulfinate		

Maxcem Elite	Resin matrix: bis-phenol-A-diglycidylmethacrylate, glycerol dimethacrylate, glycerophosphoric acid dimethacrylate	Transparent 4619346	Kerr, Orange, USA
	Filler: 67wt% content, 3.6*μ*m mean particle size, Barium aluminoborosilicate glass		

Multilink Speed	Resin matrix: dimethacrylate, 2-hydroxyethyl methacrylate, acid monomers	S05050	Ivoclar-vivadent, Schaan, Liechtenstein
	Filler: 57wt% content, 5.0*μ*m mean particle size, Barium glass fillers, ytterbium trifluoride, silicon dioxide		

## Data Availability

The data used to support the findings of this study are available from the corresponding author upon request.
